# Nanobody fusion enhances production of difficult-to-produce secretory proteins

**DOI:** 10.1016/j.jbc.2025.108292

**Published:** 2025-02-12

**Authors:** Runchuan Yan, Yan Zhang, Hui Zhang, Jiyan Ma

**Affiliations:** 1 College of Biological Sciences, China Agricultural University, Beijing, China; 2 Beijing Institute for Brain Research, Chinese Academy of Medical Sciences & Peking Union Medical College, Beijing, China; 3 Chinese Institute for Brain Research, Beijing, China; 4 State Key Laboratory of Cognitive Neuroscience and Learning, Beijing Normal University, China

**Keywords:** fusion protein, intracellular trafficking, intrinsically disordered protein, nanobody, prion, protein secretion

## Abstract

Secretory protein expression in mammalian cells is widely used in various fields, including biomedical research and biopharmaceutical production. However, achieving high-level expression of certain secretory proteins/peptides can be challenging. The naturally occurring N1 fragment of the prion protein is one of these difficult-to-produce secretory proteins, which hinders our understanding of its biological functions and limits its potential as a therapeutic molecule. To improve N1 production, we screened several well-folded protein domains and found that fusing N1 with a camelid nanobody (Nb) improved its translocation into the endoplasmic reticulum and significantly enhanced its secretion. Nb fusion does not alter the translocation mechanism, which remains dependent on the Sec61–Sec62–Sec63 complex. This approach also resulted in a significant increase in N1 production in the mouse brain using recombinant adeno-associated virus. Furthermore, fusing Nb to another unstructured protein, Shadoo (without glycosylphosphatidylinositol anchor), or a peptide hormone, somatostatin, also greatly increased their production, demonstrating the applicability of this approach to other proteins and peptides. The enhancement of N1 production is comparable or better than Fc fusion, and the effect is observed with all tested camelid Nb but not with a shark Nb and to a lesser extent with a human immunoglobulin heavy chain variable region. Importantly, the Nb in the fusion protein retained its antigen-binding capability, paving the way for the development of a dual-functional protein. Collectively, we present a novel strategy for enhancing the production of secretory proteins, which holds great promise in creating functional biological molecules for a wide range of applications.

Mammals produce a wide variety of secreted proteins that undergo processing, folding, and modification within the secretory pathway (1). This pathway is extensively utilized in the biomedical field, including the production of biotherapeutics by the pharmaceutical industry (2). In addition to intact secretory proteins, proteolytic cleavage of these proteins leads to the formation of biologically active peptides, such as neuropeptides or peptide hormones, which modulate cell–cell communication and physiological functions (3).

Similar to neuropeptides, the N1 fragment is a proteolytic product of the prion protein (PrP), a cell surface–localized glycosylphosphatidylinositol (GPI)-anchored protein well known for its involvement in prion disease (4). Under physiological conditions, PrP can be cleaved between amino acids 110 and 111, resulting in two fragments: a well-folded globular C1 fragment that remains on the cell membrane *via* its GPI anchor and an intrinsically disordered N1 fragment that is released into the extracellular space (5). The N1 fragment has been reported to have a variety of biological activities, including mediating communication between microglia and surrounding cells (6), regulating neural stem cell quiescence (7), inducing large spontaneous ionic currents (8, 9), acting as a ligand for the G-protein–coupled receptor Adgrg6 to maintain the myelin sheath (10), protecting neurons against stress (11), and selectively binding toxic amyloid oligomers formed by Aβ, tau, or α-synuclein and reducing their toxicities (12, 13, 14, 15, 16, 17, 18, 19). To verify its proposed biological activities *in vivo* and test its potential as a neuroprotective agent, stable expression of N1 at sufficient levels is required. However, the expression of N1 has been challenging (20), likely due to its lack of a defined structure and propensity to misfold. As N1 is naturally unstructured, it is classified as an intrinsically disordered region (IDR) (21). Previous studies have shown that this type of IDR faces difficulties in entering the endoplasmic reticulum (ER), which hampers its secretion from the cell (22, 23). Developing an approach to overcome these obstacles and improve N1 production, including enhancing its intracellular trafficking and secretion, would allow us to investigate its potential functions in a relevant biological system.

Generally, the secretion of IDRs occurs alongside structured regions, highlighting the importance of structured regions in facilitating IDR secretion. For instance, while full-length PrP is highly expressed in the brain, the expression of N1 alone, the unstructured portion of PrP, is so low that it cannot be used to test its biological activity (20). The Fc domain of immunoglobulin is commonly employed as a structural chaperone to stabilize fusion proteins and extend their half-life (24, 25, 26). However, Fc-fusion proteins can interact with Fc receptors and may elicit additional activities such as antibody-dependent cell-mediated cytotoxicity or complement-mediated cytotoxicity. While these activities can be beneficial in some cases, they can also be undesirable side effects in other cases (27). In addition, the Fc domain can dimerize or oligomerize, potentially adversely affecting the effectiveness of fusion proteins (27). In the case of small peptides, the Fc domain is relatively large and may sterically hinder their actions. Moreover, Fc is not universally effective for all secretory proteins (24). Therefore, the development of an alternative approach to enhance secretory protein production without these limitations would be highly desirable.

In this study, we used the unstructured N1 fragment of PrP (88 amino acids) as a model protein to investigate new strategies to improve secretory protein production. Since full-length PrP, comprising the unstructured N1 and well-folded C1 fragments, is highly expressed *in vivo*, we hypothesized that a well-folded C-terminal domain would facilitate the production of N1. We selected nanobody (Nb) because its size is similar to that of the C-terminal globular region of PrP. Additionally, Nb is well folded, is highly stable, and has been utilized as a chaperone for the structural analysis of intrinsically disordered proteins (28, 29, 30). To minimize interaction with other endogenous proteins, we chose an anti–green fluorescent protein (GFP) Nb (31, 32, 33) for our analyses. In addition, we selected a panel of well-folded and stable protein domains (Fig. 1*A*), including the human Pin1 WW domain (WW) (34, 35, 36), the variant-WW domain (V-WW) (37), the villin headpiece (HP35) domain (38, 39), the trp-cage (TC) (40, 41, 42, 43), and the N-terminal domain of ribosomal protein L9 (NTL9) (44, 45, 46). We also included the prion-like domain of ZIP10 (ZIP) because of its structural similarity to the C-terminal region of PrP (47). We observed a significant increase in the production of N1 in cultured cells and in mice when fused with Nb. Furthermore, we found that Nb fusion can also significantly improve the production of other secretory proteins or small peptides. This capability is shared among all tested camelid Nbs but not with a shark Nb and to a lesser extent with a human immunoglobulin heavy chain variable region. These results suggest that camelid Nb fusion is an alternative strategy to Fc-fusion for generating biologically active secretory proteins or peptides.

**Figure 1:**
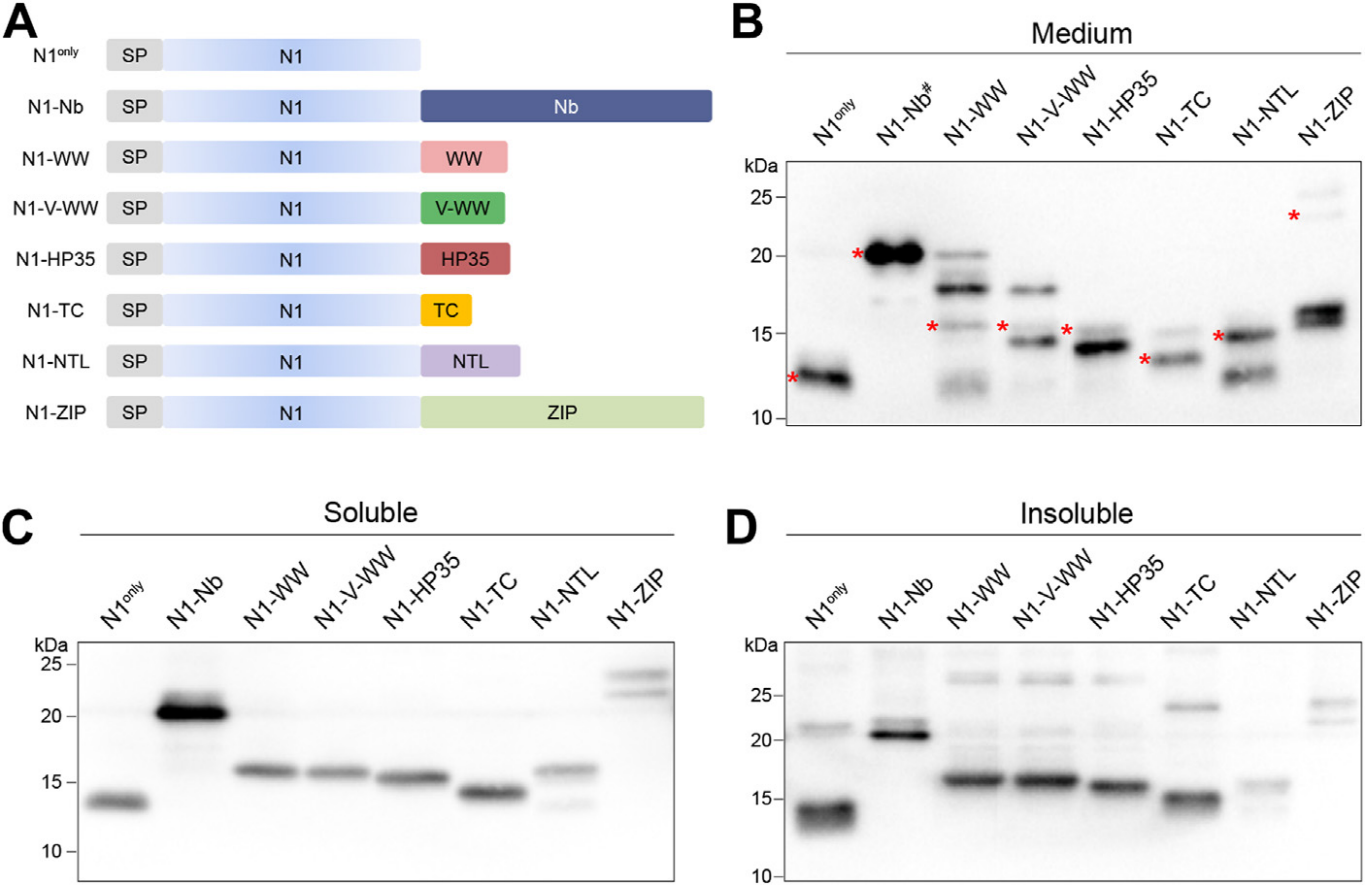
**Nb efficiently improves the production of unstructured N1 in cultured cells.***A*, schematic diagram of N1 fusion proteins. *B*, proteins in culture media were detected by Western blot analysis with 6D11 anti-PrP antibody. # Medium of N1-Nb expressing cells was diluted 375 times, whereas the other medium samples were undiluted. *Asterisks* indicate bands with expected molecular weight. *C*, fusion proteins in the soluble fraction of cell lysates were detected by Western blot analysis with 6D11 anti-PrP antibody. *D*, fusion proteins in the insoluble fraction of cell lysates were detected by Western blot analysis with 6D11 anti-PrP antibody. SP, signal peptide.

## Results

### Nb significantly improves N1 production in cultured cells

To determine how well-folded protein domains affect N1 production, we generated plasmids that express N1 fused with each domain at the C terminus (Fig. 1*A*, amino acid sequences of each domain are listed in Table S1). Culture media were collected from 293T cells transiently transfected with these plasmids, and the N1 fusion proteins in the media were detected by Western blot (WB) analysis with 6D11 anti-PrP antibody (recognizing an epitope at amino acids 93–109). Remarkably, Nb fusion drastically enhanced the secretion of N1, and level of the Nb fusion protein (N1-Nb) in the medium surpassed that of cells expressing N1 alone (N1^only^) even after being diluted 375 times (Fig. 1*B*). Densitometry analysis revealed that the level of N1-Nb was approximately 1650 times of N1^only^. This finding was confirmed by two additional replicate experiments, in which the secretion levels of N1-Nb were around 1200 times (Fig. S1, *A* and *B*) of N1^only^.

Fusion with other domains also appeared to improve production to some extent, but the levels of these N1 fusion proteins in the media were much lower than that of N1-Nb (Fig. 1*B*). Some bands migrated faster or slower than expected molecular weight, which may result from degraded and/or dimerization of degraded proteins in the cell culture media. Nevertheless, these results clearly demonstrate the significant impact of Nb fusion on improving N1 production.

To explore the influence of these domains on N1 processing within the cell, we separated cell lysates into soluble fractions (indicating correctly processed proteins in the secretory pathway) and insoluble fractions (indicating misfolded and aggregated proteins retained within cells). In the soluble fractions, all proteins migrated to positions with expected molecular weights except for N1-ZIP, which migrated as a doublet (Fig. 1*C*). In the insoluble fractions, more slower migrating bands were detected in most samples, which may indicate the presence of SDS-resistant dimers or oligomers (Fig. 1*D*). Interestingly, a band of approximately 20 kDa was observed in cells expressing N1^only^, which was not detected in media or soluble fraction, indicating that N1 is able to form an SDS-resistant insoluble dimer within the cell. Importantly, no significant difference was observed between N1^only^ and N1-Nb in the soluble and insoluble fractions, indicating that this difference could not account for the drastic increase of N1-Nb in the media. We hypothesized that the Nb fusion enhances N1-Nb production by accelerating its trafficking through the secretory pathway.

### Nb fusion enhances the translocation into the ER through the Sec61–Sec62–Sec63 complex

**Figure 2:**
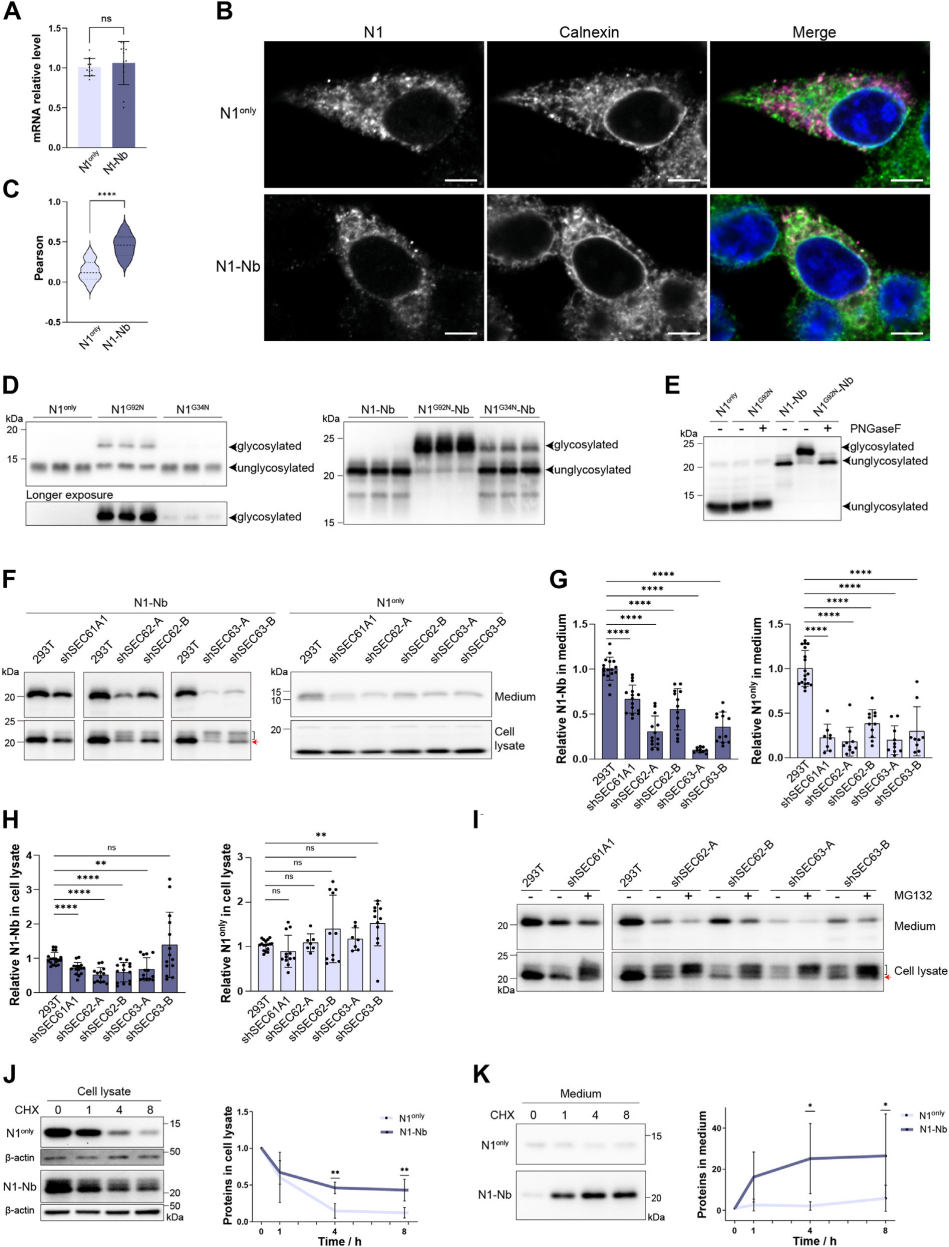
**Nb enhances the translocation of N1 into endoplasmic reticulum.***A*, RT-PCR analysis of mRNA levels of N1^only^ and N1-Nb. Statistical analysis was performed with two-tailed unpaired *t* test (n = 12, *p* = 0.5442). *B*, immunofluorescence staining of N1^only^, N1-Nb, and calnexin as indicated. The scale bar represents 5 μm. *C*, Pearson coefficients for colocalization of N1^only^ (n = 30) or N1-Nb (n = 9) with calnexin. Statistical analysis was performed using two-tailed unpaired *t* test (∗∗∗∗*p* = 0.000032). *D*, N1^only^ and N1-Nb, with or without the G92N and G34N point mutations, were detected in medium by immunoblot analysis using the 6D11 anti-PrP antibody. *E*, N1^only^ and N1-Nb in cell lysates, with or without the G92N point mutation, were treated with or without PNGase F as indicated. N1 and N1-Nb were detected by immunoblot analysis with the 6D11 anti-PrP antibody. *F*, N1-Nb and N1^only^ in the cell culture medium (*top*) and cell lysates (*bottom*) of wildtype, Sec61, Sec62, or Sec63 knockdown 293T cells were detected by immunoblot analysis using the 6D11 antibody. Bracket indicates unprocessed proteins, and red arrow indicates mature proteins. *G*, statistical analyses of N1-Nb and N1^only^ in the medium were conducted using one-tailed unpaired *t* test. The levels of N1-Nb and N1^only^ in the medium of Sec61, Sec62, or Sec63 knockdown 293T cells were compared with those of wildtype 293T cells (n ≥ 8, ∗∗∗∗*p* < 0.0001). *H*, statistical analyses of N1-Nb and N1^only^ in cell lysates were conducted using one-tailed unpaired *t* test. The levels of N1-Nb and N1^only^ in the cell lysates of Sec61, Sec62, or Sec63 knockdown 293T cells were compared with those of the wildtype 293T cells (n ≥ 7, left: ∗∗∗∗*p* < 0.0001, ∗∗*p* = 0.0038 and ^ns^*p* = 0.071 and right: ∗∗*p* = 0.0033 and ^ns^*p* > 0.05). *I*, the levels of N1-Nb in the medium and lysates of the indicated cells, with or without MG132 treatment, were determined by immunoblot analysis using the 6D11 antibody. Bracket indicates unprocessed proteins, and red arrow indicates mature proteins. *J*, transient transfected cells were treated with cycloheximide and collected at 0, 1, 4, and 8 h as indicated. N1^only^ and N1-Nb were detected by Western blot analysis with 6D11 antibody. β-actin was used as the loading control. Statistical analysis of intracellular levels of N1^only^ (n = 4) and N1-Nb (n = 6) (∗∗*p* = 0.0077 at 4 h and ∗∗*p* = 0.0089 at 8 h). *K*, media were collected at 0, 1, 4, and 8 h after cycloheximide treatment as indicated, and the presence of N1^only^ and N1-Nb was detected by Western blot analysis with 6D11 antibody. Statistical analysis of levels of N1^only^ (n = 4) and N1-Nb (n = 6) (∗*p* = 0.0191 at 4 h and ∗*p* = 0.0423 at 8 h). Statistical analyses of *J* and *K* were performed with two-way ANOVA followed by Bonferroni's multiple comparisons test.

To investigate the cellular mechanism, we analyzed the influence of Nb fusion on transcription levels and the intracellular processing of N1, including its translocation into the ER, intracellular stability, and trafficking. We performed real-time qRT-PCR and found no statistically significant difference between the mRNA levels of N1^only^ and N1-Nb (Fig. 2*A*), indicating that the enhanced secretion is not due to increased transcription. Immunofluorescence staining was performed to evaluate the influence of Nb fusion on ER entrance. In cells expressing N1^only^ (Fig. 2*B*, top), more punctate staining of N1 was observed, which did not colocalize with ER chaperone calnexin (Pearson coefficient = 0.14, Fig. 2*C*). Nb fusion (Fig. 2*B*, bottom) reduced the amount of punctate staining and enhanced its colocalization with calnexin (Pearson coefficient = 0.45), suggesting that Nb fusion enhanced the entry of unstructured N1 into ER.

To further validate our conclusion, we generated two mutant N1 variants with point mutation at G34N and G92N, which introduced artificial glycosylation site at residues 34 and 92, respectively. Since N-linked glycosylation occurs exclusively within the lumen of ER, an increase in translocation into the ER would lead to higher levels of glycosylation. Analysis of the N1^only^ and N1-Nb variants in the cell culture medium revealed that both sites could be glycosylated; however, G92N exhibited higher efficiency in N-linked glycosylation (Fig. 2*D*), indicating that it is the preferred site (48). Since the protein levels in the medium reflected accumulation over time, we compared the N1 protein glycosylation status within the cell lysates, which indicated protein translocation into the ER but not secretion. The glycosylation of N1^G92N^ was nearly undetectable, whereas 76.2% ± 5.62% (n = 12) of N1^G92N^-Nb was glycosylated, with the N-linked oligosaccharides removable by peptide-N-glycosidase F (PNGase F) (Fig. 2*E*). These findings confirmed that the Nb fusion facilitates the entry of N1 into the ER, thereby enabling its glycosylation and subsequent secretion.

It has been shown that full-length PrP exhibits a delayed initiation of translocation into the ER, with translocation beginning at polypeptide chain lengths of approximately 170 to 207 amino acids (49). This property may account for the dramatic enhancement of ER entry by the C-terminal fusion of Nb to N1, which consists of 110 amino acids. To determine whether N1-Nb translocates into the ER *via* the same mechanism, we took advantage of the fact that delayed translocation of PrP requires a complex of Sec61, Sec62, and Sec63 (49) and created stable 293T cell lines with individual knockdowns of Sec61, Sec62, and Sec63 (Fig. S2). We found that knocking down any one of the Sec proteins resulted in significantly reduced secretion of both N1-Nb and N1^only^ into the medium (Fig. 2*F* top, and Fig. 2*G*), indicating that all three Sec proteins are required for the translocation of both N1^only^ and N1-Nb into the ER. When total cell lysates were analyzed, N1-Nb levels were significantly reduced, while the change in N1^only^ levels were less evident (Fig. 2*F* bottom, and Fig. 2*H*). Notably, in the lysates of all Sec protein knockdown cell lysates, the slower migrating unprocessed N1-Nb bands appeared increased, whereas the mature N1-Nb bands were decreased, indicating that the reduced Sec protein levels hindered the processing and translocation of N1-Nb (Fig. 2*F* bottom), resulting in increased proteasome degradation of the unprocessed proteins (Fig. 2*I*). These results support that ER translocation of N1-Nb shares the same mechanism of full-length PrP and the Nb fusion enhances the efficiency of the translocation process.

Using cycloheximide (CHX) to inhibit protein synthesis, we observed a more rapid degradation of N1^only^ compared with N1-Nb (Fig. 2*J*), indicating that a greater number of N1^only^ molecules failed to enter the ER and were degraded in the cytosol, whereas the efficient ER translocation of N1-Nb protects it from such degradation. Consistent with this interpretation, we detected little change in the amount of N1^only^ in the media during the 8-h CHX treatment but a significant increase in secreted N1-Nb (Fig. 2*K*). Notably, the levels of N1-Nb in the media appeared to rise rapidly and reach a plateau around 4-h chase, suggesting an efficient trafficking of N1-Nb through the secretory pathway.

Collectively, our results indicate that Nb fusion significantly enhances the entry of fusion protein into the ER through the Sec61–Sec62–Sec63 complex, which results in the drastic increase in N1 production in the media.

### Nb fusion improves N1 production in mouse brain

**Figure 3:**
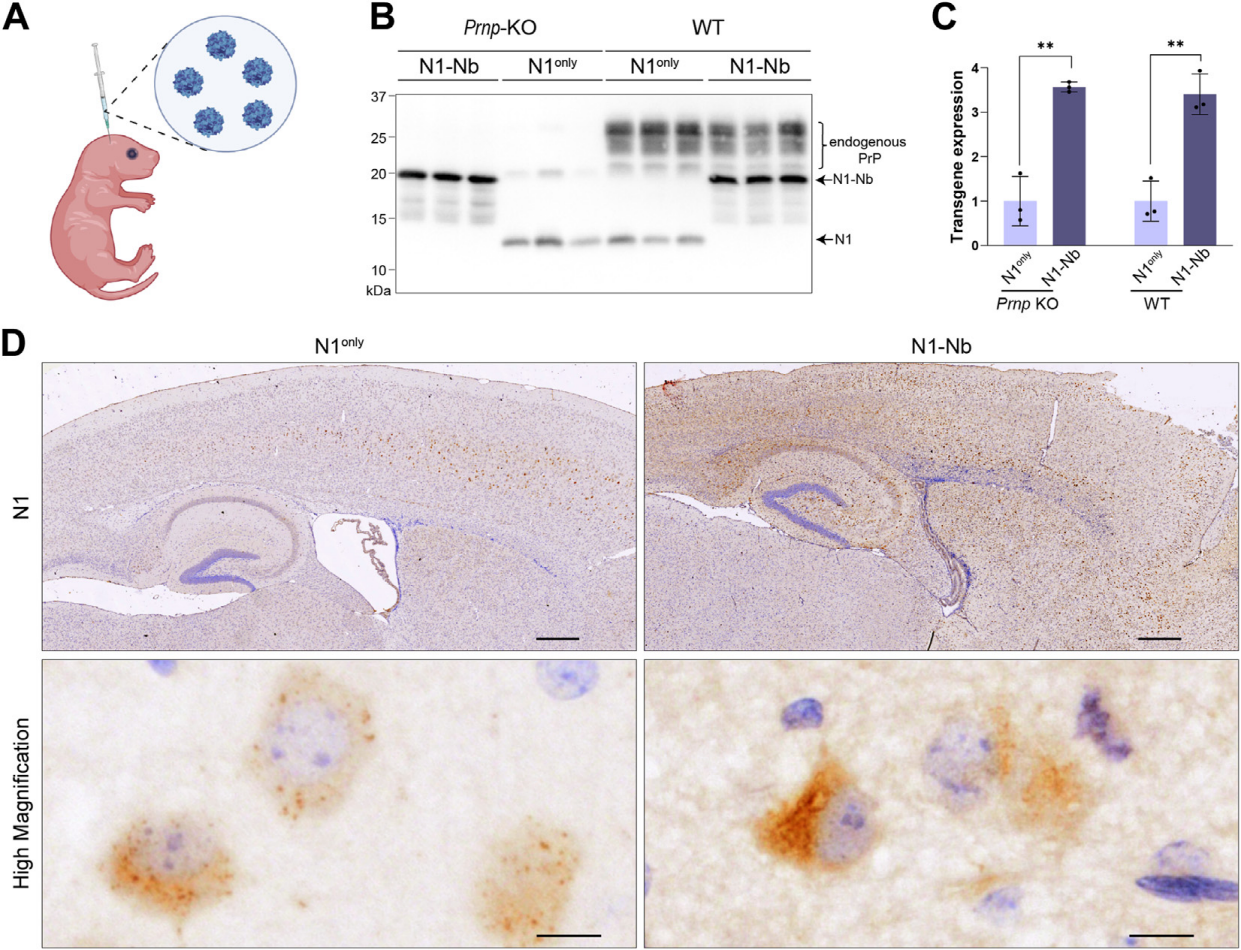
**Nb improves N1 production in mouse brain.***A*, schematic diagram illustrating intracerebroventricular injection of rAAV in newborn mice using BioRender. *B*, N1^only^ and N1-Nb in the total brain homogenates of wildtype (WT) and *Prnp* knockout (*Prnp*-KO) mice were detected by Western blot analysis with 6D11 anti-PrP antibody. Expected positions of N1, N1-Nb, and endogenous PrP were indicated. *C*, statistical analysis of N1^only^ and N1-Nb in *Prnp* KO and WT mouse brain as indicated (n = 3) (∗∗*p* = 0.00626 in KO mice and ∗∗*p* = 0.00145 in WT mice) through one-tailed unpaired *t* test. Total protein was used as the loading control. *D*, immunohistochemical staining of N1^only^ and N1-Nb in *Prnp* KO mice with 6D11 anti-PrP antibody. High-magnification images are shown in the *bottom panel*. The gamma values in all images are 0.8. Scale bars represent 1 mm in the *top panel* and 10 μm in the *bottom panel*.

To determine if Nb fusion has a similar effect *in vivo*, we packaged N1 and N1-Nb into recombinant adeno-associated viruses (rAAV) to create N1-rAAV and N1-Nb-rAAV, respectively. These viruses were delivered *via* intracerebroventricular injection in neonatal wildtype (WT) or *Prnp* knockout (KO) mice (50) (Fig. 3*A*). WB analysis revealed that levels of N1-Nb were significantly higher than those of N1^only^ in both WT and KO mice (Fig. 3, *B* and *C*). Thus, we conclude that Nb fusion improves N1 production both *in vitro* and *in vivo*.

Immunohistochemical staining was performed to compare the distribution of N1^only^ and N1-Nb *in vivo*. For this analysis, *Prnp* KO mice were used to avoid interference from endogenous PrP. Consistent with findings from WB analysis, the staining of N1-Nb was much higher than that of N1^only^ (Fig. 3*D*, top). Higher-magnification images showed more punctate staining in N1^only^-expressing cells (Fig. 3*D*, bottom), which was similar to the observations in cultured cells (Fig. 2*B*). Furthermore, the ∼20 kDa band, detected only in the insoluble fraction of N1^only^-expressing cell lysates (Fig. 1*D*), was also found in N1^only^-expressing mouse brains (Fig. 3*B*). Therefore, both WB and immunohistochemical results support the notion that a portion of N1^only^ was misfolded intracellularly and retained within the cell as aggregates.

Collectively, our findings revealed that Nb fusion significantly enhances the production of unstructured N1 in the mouse brain.

### The effect of enhancing N1 production is not limited to a particular Nb

**Figure 4:**
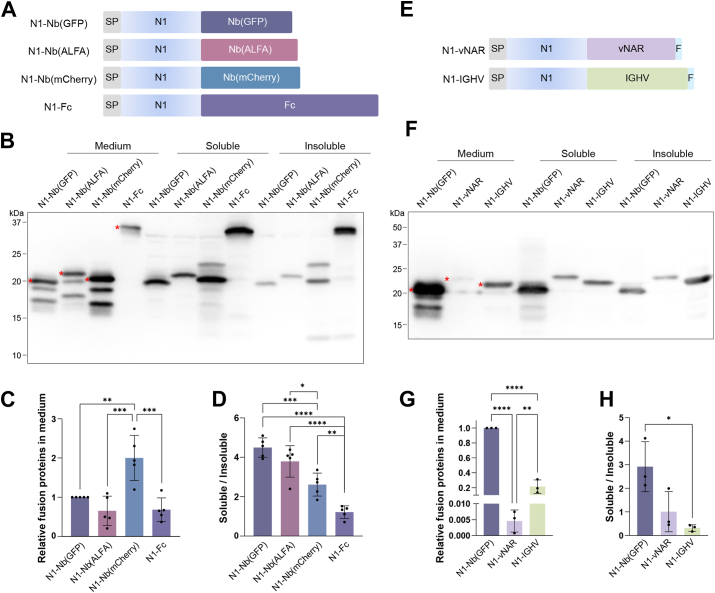
**Camelid Nb greatly improves N1 production.***A*, schematic diagram showing the N1 fused with Nb(GFP), Nb(ALFA), Nb(mCherry), or Fc. SP, signal peptide. *B*, fusion proteins in media, soluble and insoluble fractions of cell lysates were detected by Western blot analysis with 6D11 anti-PrP antibody. Bands with expected molecular weights in media were indicated by *asterisks*. Statistical analyses of fusion proteins in media (*C*) (∗∗∗*p* = 0.0002 and ∗∗*p* = 0.0033) and the soluble-to-insoluble ratios (*D*) (∗∗∗∗*p* < 0.0001, ∗∗∗*p* = 0.0005, ∗∗*p* = 0.0069, and ∗*p* = 0.0239) were carried out with one-way ANOVA (n = 5) followed by Tukey's multiple comparisons test, normalized by total protein. *E*, schematic diagram illustrating the fusion of N1 with vNAR and IGHV. *F*, fusion proteins in media, soluble and insoluble fractions of cell lysates were detected by Western blot analysis with 6D11 anti-PrP antibody. Bands with expected molecular weights in media were indicated by *asterisks*. Statistical analyses of fusion proteins in media (*G*) (∗∗∗∗*p* < 0.0001 and ∗∗*p* = 0.0064) and the soluble-to-insoluble ratios (*H*) (∗*p* = 0.0162) were carried out with one-way ANOVA (n = 3) followed by Tukey's multiple comparisons test, normalized by total protein. F, Flag tag; SP, signal peptide.

To determine whether the enhancement of N1 production is the result of the anti-GFP Nb (31, 32, 33) or a general effect of Nbs, we tested two additional Nbs. One is an Nb against a designed peptide tag ALFA, Nb(ALFA) (51), and the other is an Nb against another fluorescence protein mCherry, Nb(mCherry) (52). Mouse IgG1 Fc (Fc), a commonly used fusion partner for secretory protein expression (24), was used as a control. Plasmids expressing N1 fused with each of these partners (Fig. 4*A*) were used to transiently transfect 293T cells. WB results revealed that all of them improved N1 production (Fig. 4, *B* and *C*). Levels of N1 fused to all three Nbs in the cell culture media were comparable with or higher than that of N1-Fc, and the highest level was achieved with the N1-Nb(mCherry) fusion protein (Fig. 4*C*). When cell lysates were separated into soluble and insoluble fractions, the soluble/insoluble ratios of all N1-Nb fusion proteins were higher than that of N1-Fc (Fig. 4*D*), indicating that Nbs might be better than Fc in enhancing ER translocation and preventing misfolding and aggregation of N1.

To determine whether other single-chain antigen-binding domains have a similar effect, we tested a shark Nb, variable new antigen receptor (vNAR) (53), and a human immunoglobulin heavy chain variable region (IGHV) (54). N1 fused with vNAR or IGHV (Fig. 4*E*) was expressed in 293T cells by transient transfection. The amount of N1-vNAR in the media was very low, over 200-fold lower than that of N1-Nb (Fig. 4, *F* and *G*). IGHV performed somewhat better, but compared with N1-Nb, the level of N1-IGHV in the media was still ∼5-fold lower. Both N1-vNAR and N1-IGHV had lower soluble-to-insoluble ratios, and the difference between N1-Nb and N1-IGHV was statistically significant (Fig. 4*H*). These observations led us to conclude that camelid Nb appears to have unique advantages in enhancing N1 production.

Together, our findings suggested that camelid Nb fusion is comparable with, if not better than, Fc fusion in enhancing N1 production. Although there were differences among the three camelid Nbs tested, all of them greatly improved N1 production.

### Nb fusion significantly improves the production of other difficult-to-produce secretory proteins and peptides

**Figure 5:**
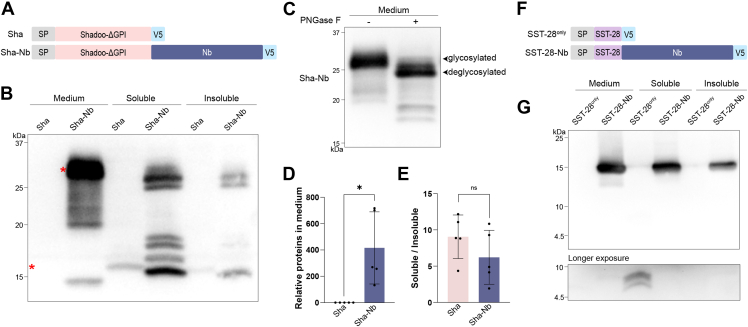
**Nb enhances the production of Shadoo-ΔGPI and SST-28.***A*, schematic illustration of Shadoo-ΔGPI (Sha) and Shadoo-ΔGPI-Nb fusion protein (Sha-Nb). *B*, Sha and Sha-Nb in media, soluble and insoluble fractions of cell lysates were detected by Western blot analysis with V5 antibody. *Asterisks* indicate the expected position of Sha and Sha-Nb in media. *C*, secreted Sha-Nb proteins with or without deglycosylation with PNGase F as indicated were detected by Western blot analysis with V5 antibody. Statistical analyses of Sha and Sha-Nb in media (*D*) (∗*p* = 0.0275) and the soluble-to-insoluble ratios of Sha and Sha-Nb in cell lysates (*E*) (^ns^*p* = 0.2222) were performed with two-tailed unpaired *t* test (n = 5), normalized by total protein. *F*, schematic depiction of SST-28^only^ and SST-28-Nb fusion protein. *G*, SST-28^only^ and SST-28-Nb in media, and soluble and insoluble fractions of cell lysates were detected by Western blot analysis with V5 antibody. The bottom panel is a longer-exposed blot to show SST-28^only^. SP, signal peptide; V5, V5 tag.

To address the question of whether this approach can be applied to secretory proteins other than N1, we tested two additional IDRs: Shadoo-ΔGPI and somatostatin (28 amino acids, SST-28). Shadoo is an unstructured GPI-anchored glycoprotein and shares some similarities with N1 (55). After removing the GPI signal sequence, Shadoo-ΔGPI is unable to enter the secretory pathway and is retained within the cell (56, 57, 58). We constructed two plasmids: Shadoo-ΔGPI plus a V5 tag (Sha) and Shadoo-ΔGPI fused with Nb(GFP) plus a V5 tag (Sha-Nb) (Fig. 5*A*). Without its GPI anchor, almost no Sha was detected in the media (Fig. 5, *B* and *D*). However, when it was fused with an Nb, a high amount of Sha-Nb was found in the media. The band of Sha-Nb in the media appeared to be higher and broader than that of intracellular Sha-Nb, indicating that it was glycosylated. Using PNGase F, we confirmed that the secreted Sha-Nb was modified with N-linked oligosaccharides (Fig. 5*C*). The soluble-to-insoluble ratio of Sha-Nb was not significantly higher than that of Sha (Fig. 5*E*), consistent with the idea that Nb fusion enhances secretory protein production by facilitating its entry into the ER and accelerating its trafficking through the secretory pathway.

To determine whether Nb fusion can improve the production of short peptides, we chose somatostatin (28 amino acids, SST-28), a peptide hormone that is naturally produced through proteolytic cleavage and secreted. Without the structured prodomain, the short and unstructured SST-28 is difficult to produce (23, 56). We compared SST-28 plus a V5 tag (SST-28^only^) with SST-28 fused with an Nb plus a V5 tag (SST-28-Nb) (Fig. 5*F*). After transient transfection in 293T cells, a large amount of SST-28-Nb was readily detectable in the media, yet no detectable SST-28^only^ was found in the media even after a prolonged exposure (Fig. 5*G*). Only in the soluble fraction of cell lysates, SST-28^only^ could be detected after overexposure. Notably, two bands were detected, and the faster migrating band had the expected molecular weight of processed SST-28, while the slower migrating band had a molecular weight of SST-28 plus the signal peptide (Fig. 5*G*, bottom), suggesting that a significant portion of SST-28^only^ failed to enter the ER. In contrast to SST-28^only^, a significant amount of SST-28-Nb was detected in both soluble and insoluble fractions.

Altogether, our results clearly showed that Nb fusion can improve the production of other secretory proteins or peptides and the secretory proteins are able to undergo proper posttranslational modifications.

### Nb remains functional in fusion protein

**Figure 6:**
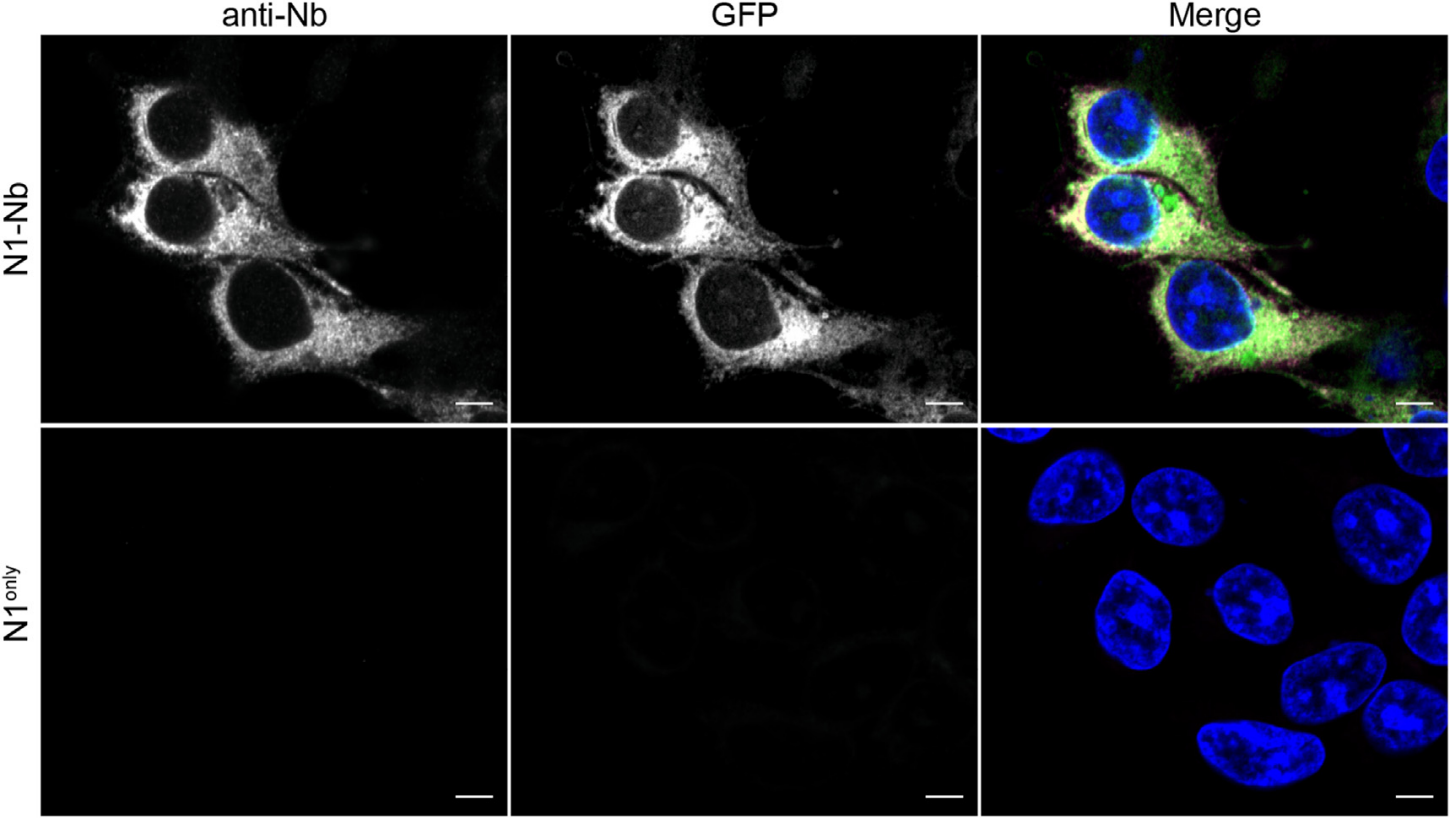
**Nb retains the antigen recognition capability in fusion protein.** The N1-Nb fusion protein and N1^only^ were detected by immunofluorescent staining with the VHH antibody that recognizes Nb. Recombinant GFP was added to show that the Nb(GFP) in fusion was able to bind GFP. The scale bar represents 5 μm.

To determine whether the Nb retains its antigen recognition capability in the fusion protein, we performed dual labeling immunofluorescence on cells expressing N1-Nb(GFP) or N1^only^ only. An anti-Nb antibody was used to label the Nb, and recombinant GFP was added to determine if the Nb in the fusion protein could still recognize and bind GFP. Figure 6 showed that, in the N1-Nb group, nearly all Nb-positive signals colocalized with GFP, suggesting that the Nb in the fusion protein retained its binding capability. In contrast, there was hardly any signal of either Nb or GFP in the N1^only^ group.

## Discussion

High-level expression of secretory proteins or peptides is generally difficult, facing challenges such as misfolding, inability to enter the secretory pathway, or proteolytic degradation. This is particularly problematic when the secretory protein is intrinsically disordered (23, 56). Fc fusion has been the classic strategy to improve the production of secretory proteins, but as mentioned earlier, this strategy has several limitations. Here, we show that Nb fusion can significantly improve the production of secretory proteins or short peptides and the effect of Nb fusion is comparable with, if not better than, Fc fusion. Since Nb is smaller and its only biological activity is antigen binding, Nb fusion achieves this effect without any undesired activities related to Fc. Furthermore, the fact that Nb in the fusion protein retains its antigen-binding capability opens the possibility of targeting fusion proteins to desired tissues, cells, or molecules, which would greatly enhance the biological activities of these fusion proteins. We envision a broad application of this strategy in biomedicine, including the development of novel therapeutic agents, biomolecule production, and investigations into the roles of secretory proteins/peptides in relevant biological systems.

Nb is the variable region of natural heavy chain antibodies found in camelids or sharks, which has a small size and remarkable stability, making it suitable for various applications including imaging, drug development, and therapeutics (59, 60). Here, we expanded its application to improving the production of secretory proteins. Since Nb can be selected through *in vitro* screening, it offers an opportunity to target the activity of secretory proteins to virtually any type of cells or molecules. The three Nbs tested in this study all showed excellent activity in improving secretory protein production, although we did observe some variabilities among them. One possible reason for this could be the inherent stability differences among these Nbs. However, this potential drawback can be addressed by implementing *in vitro* evolution and selection of Nb (61, 62). Consistent with this explanation, shark vNAR has a different structure than camelid Nb (63), failed to improve N1 production. IGHV is a human immunoglobulin heavy chain variable region that was selected based on its similarity to camelid Nb (54). Although it is highly soluble and stable, IGHV is not a natural single-chain molecule and is structurally different from Nb (64). Therefore, it is not surprising that it can only partially enhance the production of the N1 fragment. These observations led us to conclude that the unique properties of camelid Nb are important for improving the production of unstructured secretory proteins.

The N1 fragment used in our study is an intriguing molecule with numerous proposed biological activities, ranging from neuroprotection to neurotoxicity (6, 7, 8, 9, 10, 11, 12, 13, 14, 15, 16, 17, 18, 19). However, due to its lack of structure and high propensity to misfold (65), sustained high-level expression of this molecule *in vivo* has proven difficult (20). The strategy developed here successfully expressed N1 fragment *in vivo*. Compared with the *in vitro* effect, the enhancement of N1 production *in vivo* is not as high, which is probably due to the complicated *in vivo* environment. Given that a significant portion of N1^only^ were misfolded and retained inside cells, the 3.5-fold increase in N1 levels by Nb fusion is much higher than previously achieved by transgenic mice (20), which will allow us to test the biological activities of N1 *in vivo*.

Besides its impact on the study of PrP, it is noteworthy that Nb fusion is capable of significantly increasing the production of Shadoo-ΔGPI and SST-28 in media by hundreds and thousands of times, suggesting that this strategy can be applied to generate sufficient amount of other secretory proteins for their intended usages.

Our data indicate that the mechanism by which Nb fusion enhances N1 production is through a significant increase in the translocation of N1 into the ER. The translocation of proteins into the ER is a complex process that involves multiple pathways (66, 67) and the assembly of various protein complexes during translocation (49). The translocation of PrP into the ER has been shown to have a delayed initiation and require a Sec61–Sec62–Sec63 complex (49, 68). We have found that the same complex is required for the translocation of N1-Nb, suggesting that the fusion of Nb does not alter the molecular pathways for translocation but rather enhances the efficiency of the process. One theory attributes this effect to the length of the protein (69, 70), which could explain the effect based on increased length of N1-Nb. However, this theory is incompatible with the observation that, despite ZIP being longer than Nb, it does not improve N1 production. Given that numerous studies have shown that the presence of a structured domain facilitates the translocation of the unstructured secretory protein/peptide into the ER (22, 23, 56, 58, 71) and considering that Nb is well known for its tightly packed structure, along with our findings that multiple camelid Nbs—but not vNAR or IGHV—enhance N1 production, a more plausible explanation could be that the presence of structured or partially structured camelid Nb during translocation enhances the process. This explanation is also consistent with previous findings that a distal nascent peptide chain can modulate protein translocation into the ER (72, 73). Overall, our study clearly demonstrates that Nb fusion is an alternative to Fc fusion for enhancing the production of secretory proteins. This highly effective and customizable approach will have broad applications in biomedical research and clinical usage.

## Materials and methods

### Plasmid construction

The vector pAAV-CAG-tdTomato was obtained from the Vector Core at Chinese Institute for Brain Research, Beijing (CIBR) and various expression fragments (listed in Table S1) were used to replace the tdTomato coding sequence. All transgene expressions were under the control of the CAG promoter. PCR primers and templates used in this study are listed in Table S2. All synthesized DNA fragments and primers were purchased from Azenta Life Sciences (Suzhou, China). Reagents and kits for PCR amplification (cat# AP221-11) and DNA ligation (cat# CU201-02), and the Trans5α cells for transformation (cat# CD201-02) were purchased from TransGen Biotech Co., Ltd (Beijing, China). The knockdown vectors targeting Sec61, Sec62, and Sec63 were constructed on the pLKO.1, which was provided by the CIBR Vector Core. These vectors were prepared by digesting with *Age* I (cat# R3552L, NEB) and *Eco* RI (cat# R0101S, NEB) enzymes, followed by ligation of annealed shRNA primers using T4 DNA ligase (cat# FL101-01, TransGen). The shRNA sequences are as follows:

shSEC61A1: GCTGGCGCCAAGATAATTGAA;

shSEC62-A: CCAGGAAATCATGGAACAGAA;

shSEC62-B: GAAGTTGGTGAACCATCTAAA;

shSEC63-A: GTGGTATCGTTTACGGTTATT;

shSEC63-B: GCAAACAATGGCTGAAGTATT.

N1-rAAV and N1-Nb-rAAV were packaged by the Vector Core at CIBR.

### Cell culture and transfection

Human kidney 293T cells were cultured in Dulbecco's modified Eagle's medium (DMEM) (cat# C11995500BT, Thermo Fisher Scientific) supplemented with 10% fetal bovine serum (cat# SE100-B, Vistech) and 1% penicillin/streptomycin (PS, cat# 15140122, Thermo Fisher Scientific). Cells tested negative for mycoplasma. Stable cell lines with knockdown of Sec61, Sec62, and Sec63 were established by infecting 293T cells with lentivirus expressing shRNAs. Twenty-four hours post infection, the medium was replaced and the cells were cultured for an additional 48 h prior to the addition of 2 μg/ml puromycin (cat# ST551-10mg, Beyotime) for a subsequent 48-h treatment. For transfection, cells were seeded in a six-well plate 1 day prior and a mixture of 100 μl DMEM containing 2 μg plasmid and 2 μl Neofect reagent (cat# TF201201, Neofect Biotech) was added dropwise to each well. After 24 h, the medium was replaced with a serum-free DMEM medium containing 1% PS. For MG132 treatment, 5 μM of MG132 (cat# 474790, Merck) was added to the aforementioned serum-free DMEM medium containing 1% PS. Following 24-h incubation, the medium was collected to analyze the amount of secreted protein. Cells were lysed on ice with cell lysis buffer (10 mM Tris–HCl, pH 8.0, 100 mM NaCl, 0.5% Triton X-100, and 0.5% sodium deoxycholate and 1 mM phenylmethylsulfonyl fluoride) and centrifuged at 15,060*g* at 4 °C for 30 min to separate insoluble proteins (pellet) from soluble proteins (supernatant). For stable cell lines with knockdown of Sec61, Sec62, and Sec63, cells were lysed on ice with RIPA buffer (cat# P0013B, Beyotime). For CHX treatment, cell culture medium was replaced with DMEM supplemented with 50 μg/ml CHX and 1% PS 24 h after transient transfection. Cells and media were collected at indicated time points, and levels of N1^only^ and N1-Nb were determined by Western blot analyses.

### Deglycosylation

Cell culture medium or cell lysates was collected and deglycosylation reactions were carried out with PNGase F (cat# P0704L, NEB) following manufacturer’s protocol.

### Western blot analysis

Secreted, soluble, and insoluble proteins were separated with 14% SDS-PAGE, except for N1^only^ in the medium in Fig. 2*F*, which was separated with 10% SDS-PAGE. Additionally, the Sec61 samples in Fig. S2 were separated with a 14% SDS-PAGE without the stacking gel. A 16% Tricine gel (cat# EC66952BOX, Thermo Fisher Scientific) was used for the analysis of SST-28^only^ and SST-28-Nb. Western blot was performed as described (74), and primary antibodies used were 6D11 monoclonal anti-PrP antibody (1:2000, cat# 808003, BioLegend), V5 monoclonal antibody (1:2000, cat# R960-25, Thermo Fisher Scientific), anti-β-actin (1:2000, cat# A1978, Merck), Sec61 (1:1000, cat# ab183046, Abcam), Sec62 (1:1000, cat# ab140644, Abcam), and Sec63 (1:10000, cat#A305-084A, Bethyl Laboratories). Blots were developed using peroxidase-conjugated secondary antibody Goat Anti-Mouse (1:5000, cat# SA00001-1, Proteintech) or Goat Anti-Rabbit (1:5000, cat# SA00001-2, Proteintech) and ECL reagent (cat# 180-5001, Tanon) and imaged by using an iBright imaging system (FL1000, Thermo Fisher Scientific). Most of the protein levels were normalized using total protein, which was labeled by Coomassie Brilliant Blue (cat# C8430, Solarbio) staining (Fig. S3), except for Figure 2, *J* and *K*, where β-actin was used for analyses. The numbers on the left or right in each blot refer to molecular size markers (size in kDa). Mouse brains were harvested, and a 10% (w/v) brain homogenate was prepared with PBS. For Western blot analysis, brain homogenate was lysed by adding a 10x lysis buffer (5% Triton X-100 and 5% sodium deoxycholate in PBS), separated by 14% SDS-PAGE. 6D11 antibody (1:2000) was used as primary antibody. Western blot analyses of mouse brain homogenates were normalized by total protein.

### Immunofluorescence staining

293T cells were seeded in confocal dishes (cat# 801001, NEST) precoated with Poly-D-lysine (cat# P7280, Merck) 1 day prior to transfection. Twenty-four hours after transfection, cells were washed twice with PBS, fixed with ice-cold methanol for 10 min at room temperature, and washed three times with PBS. Cells were blocked with 5% bovine serum albumin at room temperature for 1 h and incubated at 4 °C overnight with the following primary antibodies: 6D11 (1:200, cat# 808003, BioLegend), Calnexin (1:100, cat# ab219644, Abcam), and VHH (1:200, cat# A01860, GenScript) or incubated with recombinant GFP (10 ng/μl, cat# P7410, Beyotime) as indicated. Afterward, cells were washed five times with PBS and incubated with a secondary antibody at room temperature for 1 h. The secondary antibodies used in this study were donkey anti-goat IgG (H+L) labeled with Alexa Fluor Plus 647 (1:1000, cat# A32849, Thermo Fisher Scientific), donkey anti-mouse IgG (H+L) labeled with Alexa Fluor 568 (1:1000, cat# A10037, Thermo Fisher Scientific), and goat anti-rabbit IgG (H+L) labeled with Alexa Fluor Plus 647 (1:1000, cat# A32733, Thermo Fisher Scientific). DAPI (1:3000, cat# 62248, Thermo Fisher Scientific) was used to stain nuclei. After incubation, cells were washed five times with PBS and imaged with an LSM880 confocal microscope (ZEISS). The colocalization pearson coefficient was analyzed using the colocalization module in ZEN (ZEISS) software.

### RT-PCR

RNA was isolated with Trizol (cat# 15596026, Thermo Fisher Scientific) following manufacturer’s protocol. Isolated RNA was diluted to 200 ng/μl and subjected to reverse transcription using HiScript III All-in-one RT SuperMix Perfect for qPCR kit (cat# R333-01, Vazyme) according to manufacturer’s protocol. Real-time qRT-PCR was performed using the Taq Pro Universal SYBR qPCR Master Mix (cat# Q712-02, Vazyme). GAPDH was used as an internal control with forward primer 5′AATCCCATCACCATCTTCCA3′ and reverse primer 5′TGGACTCCACGACGTACTCA3′. Primers for N1^only^ and N1-Nb detection were forward primer 5′ATGGTGGTAGTTGGGGTCAG3′ and reverse primer 5′TGCTTGAGGTTGGTTTTTGG3′. All primers were synthesized by Azenta Life Sciences (Suzhou, China).

### Intracerebroventricular injection of newborn mice

C57BL/6J mice were purchased from the Laboratory Animal Resource Center in CIBR. *Prnp* KO mice were created by BRL Medicine Inc (Shanghai, China) and characterized previously (50). Both sexes of mice were used in this study. All mouse experiments and procedures were approved by the animal welfare and research ethics committee of CIBR (Protocol No. CIBR-IACUC-039). For rAAV injection, newborn mice were anesthetized with ice and intracerebroventricular injection was performed as previously described (50), with a total of 3 × 10^10^ gc/pup injected bilaterally.

### Immunohistochemistry

Mouse brain was fixed in 10% neutral formalin, followed by dehydration through an alcohol gradient, embedding in paraffin, and cutting into 5-μm thick sections. Slides were then baked at 65 °C for 45 min. After rehydration with a gradient of alcohol, sections were subjected to antigen retrieval treatment, blocked with 5% bovine serum albumin for 1 h, incubated overnight with the primary antibody 6D11 (1:200), followed by a 1-h incubation with the horseradish peroxidase–labeled hypersensitive goat anti-mouse polymer (cat# PV-8000, ZSGB-Bio). After dehydration and sealing steps, stained slides were imaged with the VS120 Virtual Slide Microscope (Olympus).

### Statistical analysis

Column or violin charts were generated with GraphPad Prism 10.1.2. Error bars in all figures represent the standard deviation (SD). Each “n” value represents independent biological replicate, except for the “Pearson coefficient” n value, which accounts for individual cells from two independent experiments. Statistical significances are indicated as ∗∗∗∗ represents *p* < 0.0001, ∗∗∗ represents *p* < 0.001, ∗∗ represents *p* < 0.01, ∗ represents *p* < 0.05, and "ns" indicates no significant difference.

## Data availability

All data are contained within the article. Further inquiries can be directed to the authors.

## Supporting information

This article contains supporting information.

## Ethical approval

All animal experiments were approved by the Animal Welfare and Research Ethics Committee of Chinese Institute for Brain Research (Protocol No. CIBR-IACUC-039).

## Conflict of interest

A patent application has been filed relating to aspects of the study described in this article. J. M. and R. Y. are listed on the patent application. The other authors declare no competing interests.
