# Supplementary material for: Nanobody fusion enhances production of difficult-to-produce secretory proteins
Source: J Biol Chem. 2025 Feb 12;301(3):108292. doi: 10.1016/j.jbc.2025.108292 (PMC11930436; doi:10.1016/j.jbc.2025.108292)
Supplement: Supporting Information [file mmc1.docx]

**Nanobody fusion enhances production of difficult-to-produce secretory proteins**

Runchuan Yan^1,2,3✝^, Yan Zhang^1,2,3✝^, Hui Zhang^2,3,4^, Jiyan Ma^2,3^*

Material included:

Supplementary Figure S1: Nb efficiently promotes the production of unstructured N1 in cultured cells.

Supplementary Figure S2: Relative expression levels of Sec61, Sec62, and Sec63 in stable cell lines.

Supplementary Figure S3**:** The original blots used for immunoblot analyses in each figure are indicated.

Supplementary Table S1: Amino acid sequences of proteins and peptides used in this study.

Supplementary Table S2: Primers and templets used for PCR amplification.

**Supporting Information**

Supplementary Figure S1


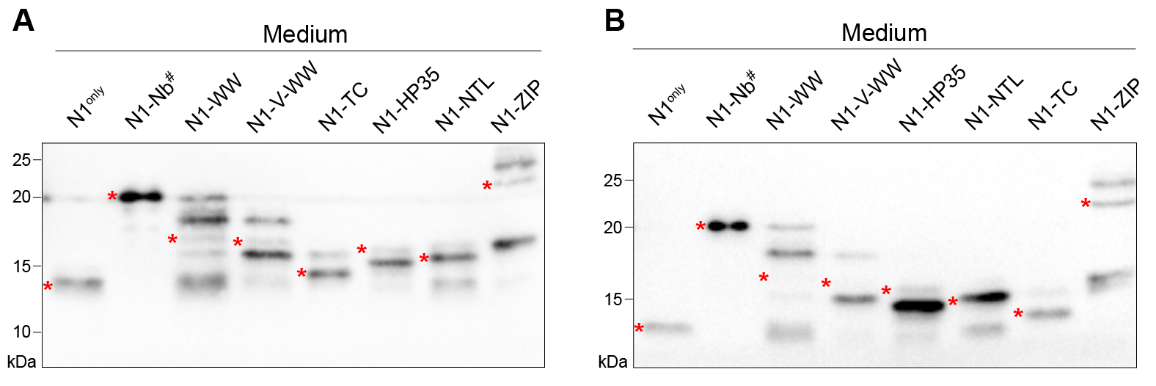
**Fig. S1.** Nb efficiently promotes the production of unstructured N1 in cultured cells. Proteins in culture media were detected by western blot analysis with 6D11 anti-PrP antibody. # Medium of N1-Nb expressing cells was diluted 500 times **(A)** and 250 times **(B)** respectively. All other media were undiluted. Asterisks indicate bands with expected molecular weight.

Supplementary Figure S2


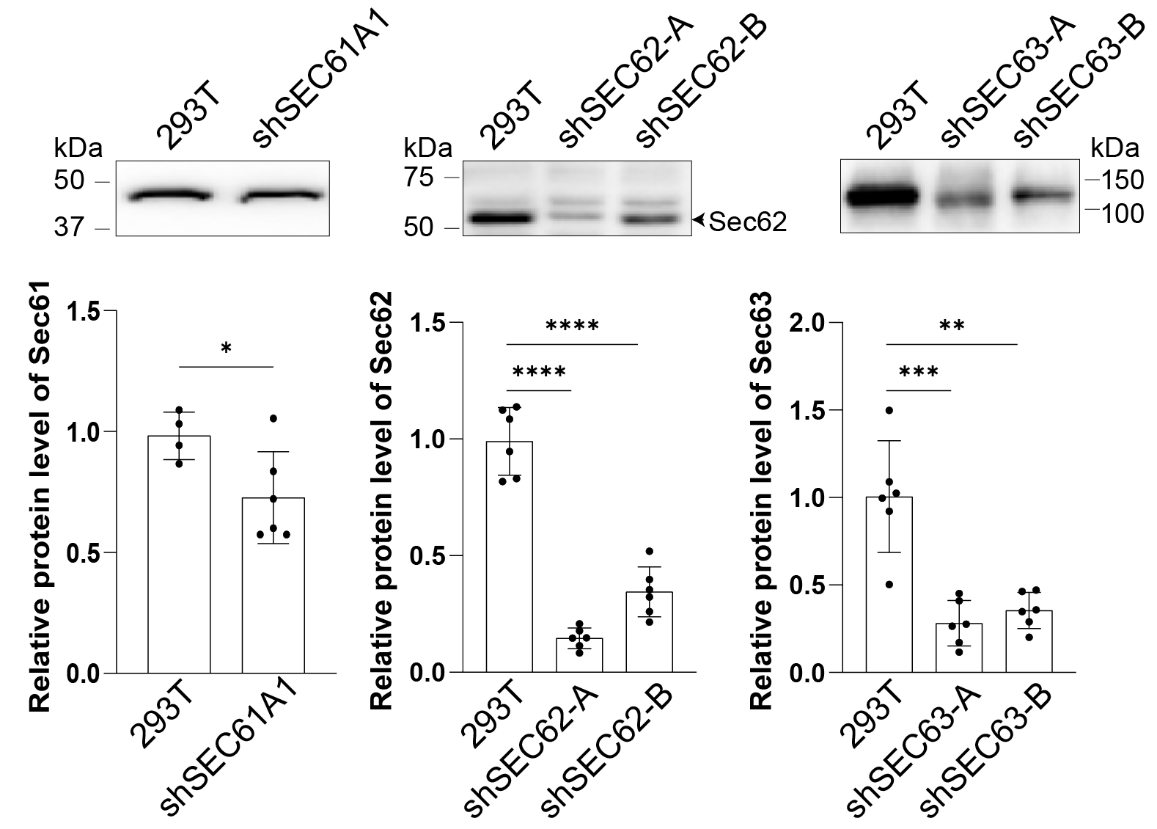


**Fig. S2.** Relative expression levels of Sec61, Sec62, and Sec63 in stable cell lines. Statistical analysis was performed using one-tailed unpaired t-test in comparison with 293T cells. (n=6 for all groups except for the 293T group in the Sec61 analysis, where n=4; *P=0.012 in the Sec61 group, ****P<0.0001 in the Sec62 group, ***P=0.00079 and **P=0.0016 in the Sec63 group).

Supplementary Figure S3


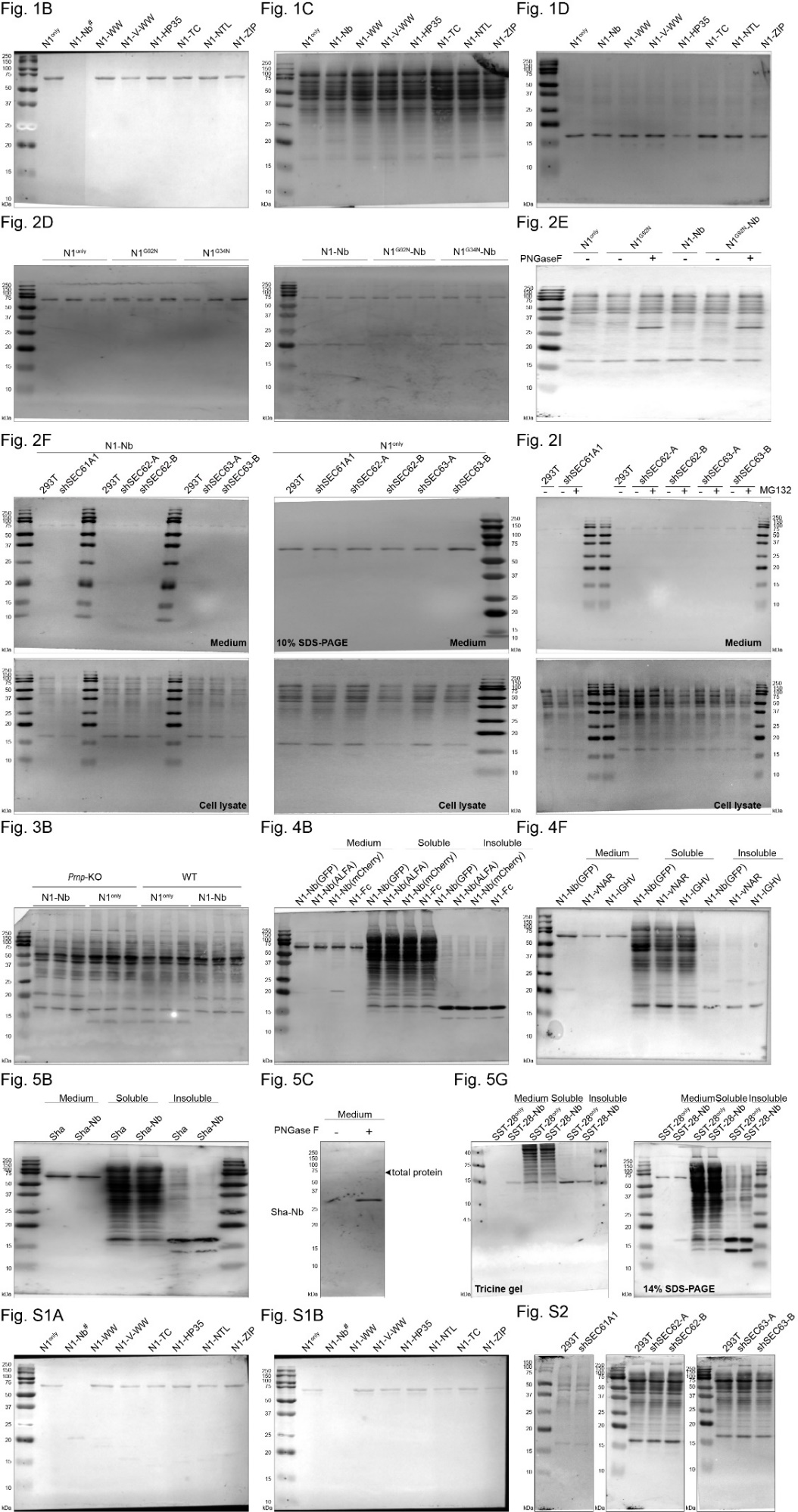


**Fig. S3.** The original blots used for immunoblot analyses in each figure are indicated. Total proteins on the original blots were stained with Coomassie Brilliant Blue. In Figure 5G, many protein bands were not visible due to the use of a Tricine gel. Therefore, the same samples were loaded onto a 14% SDS-PAGE and stained to confirm the amount of proteins loaded in each lane.

**Supplementary Table S1: Amino acid sequences of proteins and peptides**

**used in this study**

| **Name** | **Full name** | **Amino acid sequence** |
| --- | --- | --- |
| N1 |  | **MANLGYWLLALFVTMWTDVGLC**KKRPKPGGWNTGGSRYPGQGSPGGNRYPPQGGTWGQPHGGGWGQPHGGSWGQPHGGSWGQPHGGGWGQGGGTHNQWNKPSKPKTNLKH |
| Nb (GFP) | Nanobody (GFP) | VQLVESGGALVQPGGSLRLSCAASGFPVNRYSMRWYRQAPGKEREWVAGMSSAGDRSSYEDSVKGRFTISRDDARNTVYLQMNSLKPEDTAVYYCNVNVGFEYWGQGTQVTVSS |
| WW | WW domain | KLPPGWEKRMSRSSGRVYYFNHITNASQNERPSG |
| V-WW | Variant WW domain | KLPPGWEKRMSRDGRVYYFNHITNASQNERPSG |
| HP35 | villin headpiece | LSDEDFKAVFGMTRSAFANLPLWKQQHLKKEKGLF |
| TC | Trp-cage | DAYAQWLKDGGPSSGRPPPS |
| NTL | NTL9^1-39^ | MKVIFLKDVKGMGKKGEIKNVADGYANNFLFKQGLAIEA |
| ZIP | ZIP10 PL | HSGHELGHGHQELDPDNEGELRHTRKREAPHVRKSAIYSTPSHKDQSEDDRQHECLNVTQLLKHFGLGPNSPISPDLFTYLCPALLYQIDSRLCIEHFDKLLVEDLNKDKT |
| Nb (ALFA) | Nanobody (ALFA) | VQLQESGGGLVQPGGSLRLSCTASGVTISALNAMAMGWYRQAPGERRVMVAAVSERGNAMYRESVQGRFTVTRDFTNKMVSLQMDNLKPEDTAVYYCHVLEDRVDSFHDYWGQGTQVTVSS |
| Nb (mCherry) | Nanobody (mCherry) | VQLVESGGGLVQAGGSLRLSCATSGFTFSDYAMGWFRQAPGKEREFVAAISWSGHVTDYADSVKGRFTISRDNVKNTVYLQMNSLKPEDTAVYSCAAAKSGTWWYQRSENDFGSWGQGTQVTVS |
| Fc | mouse mIgG1 Fc | GCKPCICTVPEVSSVFIFPPKPKDVLTITLTPKVTCVVVDISKDDPEVQFSWFVDDVEVHTAQTQPREEQFNSTFRSVSELPIMHQDWLNGKEFKCRVNSAAFPAPIEKTISKTKGRPKAPQVYTIPPPKEQMAKDKVSLTCMITDFFPEDITVEWQWNGQPAENYKNTQPIMDTDGSYFVYSKLNVQKSNWEAGNTFTCSVLHEGLHNHHTEKSLSHSPGK |
| vNAR | Variable new antigen receptor | ERVEQTPTTTTKEAGESLTIDCVLKGSSCALGSTYWYFTKKGATKKASLSTGGRYSDTKNTASKSFSLRISDLRVEDSGYYHCEAYKGGMEAVIWNCDSYIEGGGTAVTVK |
| IGHV | Human immunoglobulin heavy chain variable region | EVQLVESGGGLVQPGGSLRLSCAASGFTVGGGGSSNYMSWVRQAPGKGLEWVSVIYSGGGGGSTYYADSVKGRFTISRDNSKNTLYLQMNSLRAEDTAVYYCARLRDGFNNGFDYWGQGTLVTVSS |
| Sha | Shadoo-ΔGPI | **MNWTAATCWALLLAAAFLCDSCSA**KGGRGGARGSARGVRGGARGASRVRVRPAPRYGSSLRVAAAGAAAGAAAGVAAGLATGSGWRRTSGPGELGLEDDENGAMGGNGTDRGVYSYWAWTSG |
| Som | Somatostatin - hormone domain | **MLSCRLQCALAALCIVLALGGVTG**SANSNPAMAPRERKAGCKNFFWKTFTSC |

Note: Signal peptide (SP) sequences are highlighted in bold.
